# Ion Transport in Plant Cell Shrinkage During Death

**DOI:** 10.3389/fcell.2020.566606

**Published:** 2020-10-19

**Authors:** François Bouteau, David Reboutier, Daniel Tran, Patrick Laurenti

**Affiliations:** ^1^Université de Paris, Laboratoire Interdisciplinaire des Energies de Demain, Paris, France; ^2^UMR 6290-IGDR Expression Génétique et Développement Faculté de Médecine, Rennes, France; ^3^Agroscope, Institute for Plant Production Systems, Conthey, Switzerland

**Keywords:** plant, cell death, ion channel, shrinkage, stress

## Introduction

Cell death (CD) is a fundamental biological process that is indispensable in all living organisms (Ameisen, 2002). Phloem differentiation, root cap, aerenchyma formation, and leaf senescence are examples of developmental CD in plants. CD also occurs in response to pathogen attacks, and to abiotic stresses such as salinity, drought or pollutants. Studies of past decades characterized CD in plant, as a surprisingly complex phenomenon with various forms and multiple pathways to achieve CD. Number of molecular actors and involved-processes such as reactive oxygen species (ROS), caspases, autophagic activities, mitochondrial dysfunction with release of cytochrome c and other apoptogenic proteins, DNA laddering, etc., have been highlighted (Reape et al., 2008; van Doorn et al., 2011). van Doorn et al. (2011) proposed a classification of plant cell deaths based on morphological characteristics. They distinguished two main classes of CD: vacuolar cell death and necrosis. During vacuolar cell death, the cell contents are removed by autophagy-like process and release of hydrolases from collapsed lytic vacuoles. Necrosis is characterized by early rupture of the plasma membrane, shrinkage of the protoplast and absence of vacuolar cell death features. They recommended abandoning terms “apoptotic-like,” because the features often cited are also found in other types of CD, whereas cytological characteristics such as formation of apoptotic bodies and phagocytosis are absent in plants. One can notice however that the absence of these morphological characteristics is obviously due to the presence of a rigid cell wall in plants. On the contrary, the team of McCabe defends the idea of an apoptotic-like CD in plants. Based on features shared with animal apoptosis, this CD shows protoplast shrinkage with a central regulatory role for the mitochondria and cell degradation mediated by proteases (Reape and McCabe, 2010). This proposition has recently been reinforced with data showing that the vacuole may carry out functions that are analogous to animal phagocytosis to remove unwanted plant cells (Dickman et al., 2017). In plant CD, the presence of the cell wall prevents cell swelling. Cell shrinkage is an event recorded in most cases, being thus one of the main hallmarks of plant CD (Reape and McCabe, 2010; van Doorn et al., 2011). Accordingly, one of the most frequent technics used to quantify plant CD is the recording of electrolyte leakage. These leakages are supposed to be due to the rupture of the plasma membrane during necrotic cell death (van Doorn et al., 2011) or by the insertion in plasma membrane of toxins with pore forming properties (Klusener and Weiler, 1999). While in animal cells the successful execution of various forms of CD relies on early activation of distinct ion channels (Okada and Maeno, 2001; Bortner and Cidlowski, 2007), the role of ion channels during plant CD remains poorly documented although there is mounting evidence that electrolyte leakages from plant cells could be mediated by plasma membrane ion channels in responses to various CD-inducing stresses.

## Ion Channel Regulations In Plant CD

An increase in K^+^ outward rectifying conductances (KORC) was recorded in response to various CD-inducing microbe-derived molecules, such as harpins (El-Maarouf et al., 2001; Haapalainen et al., 2012), deoxinivalenol (DON) (Yekkour et al., 2015) or CD-inducing ROS stress (Demidchik et al., 2010, 2014), like ozone (Tran et al., 2013a). Conductances with different activation kinetics and selectivity (Demidchik et al., 2014) are likely triggered by GORK or SKOR channels (Tran et al., 2013a; Demidchik et al., 2014), but could also be provoked by annexins, cyclic nucleotide-gated channels and ionotropic glutamate receptors. Nonetheless, the use of K^+^ channel blockers decreases KORC, CD-extent (Haapalainen et al., 2012), cell shrinkage (Yekkour et al., 2015) or even activation of metacaspases (Tran et al., 2013a), proteases and endonucleases (Demidchik et al., 2014, 2018). Recently, gork1-1 mutants lacking K^+^ efflux channel were also shown to have fewer autophagosomes compared to the wild-type plant upon ROS-induced CD (Demidchik, 2018). Activation of K^+^ efflux through KORC is supposed to result in dramatic K^+^ loss from plant cells and promotes CD (Demidchik et al., 2018) and K^+^ loss was effectively shown to be involved in tobacco cell death induced by palmitoleic acid and ceramide (Peters and Chin, 2007). Interestingly, *Nicotiana benthamiana* plants undergoing oxidative stress and transiently expressing CED-9, an anti-apoptotic gene from the bcl-2 family (Craig, 1995), are capable of preventing K^+^ efflux and maintaining intracellular K^+^ homeostasis (Shabala et al., 2007).

In response to ozone or DON, plant cells rapidly activate anion currents, followed by a delayed activation of KORC (Tran et al., 2013a; Yekkour et al., 2015), the whole ion efflux being thus transiently not electroneutral. Such increases in anion currents were also recorded with other CD-inducing microbe-derived molecules, such as harpins (Reboutier et al., 2007), fusaric acid (Bouizgarne et al., 2006), oxalic acid (Errakhi et al., 2008), cryptogein (Gauthier et al., 2007), or with ozone (Kadono et al., 2010), drought (Dauphin et al., 2001) or hydroxyl radicals (Pottosin et al., 2018). The anion currents recorded present the features of slow anion channels encoded by the slac family (Hedrich, 2012), although the instantaneous current could be carried out, to some extent, by fast activating anion channels from ALMT family (Hedrich, 2012). Either way, inhibition of anion currents with anion channel blockers reduced CD-extent (Errakhi et al., 2008; Kadono et al., 2010; Yekkour et al., 2015), vacuolar collapse and cell shrinkage (Gauthier et al., 2007; Yekkour et al., 2015). It also prevented mitochondrial dysfunction (Errakhi et al., 2008; Kadono et al., 2010), caspase-like activities (Tran et al., 2013b) and the accumulation of transcripts encoding vacuolar processing enzymes (Gauthier et al., 2007; Kadono et al., 2010), that belongs to a family of proteases displaying a caspase 1-like activity.

In plant the vacuolar collapse seems a key step in cell shrinkage but to our knowledge the role of vacuolar conductances is poorly documented. Therefore, further studies are needed to decipher the role of vacuolar channels in this process.

## Conclusion

**Figure 1:**
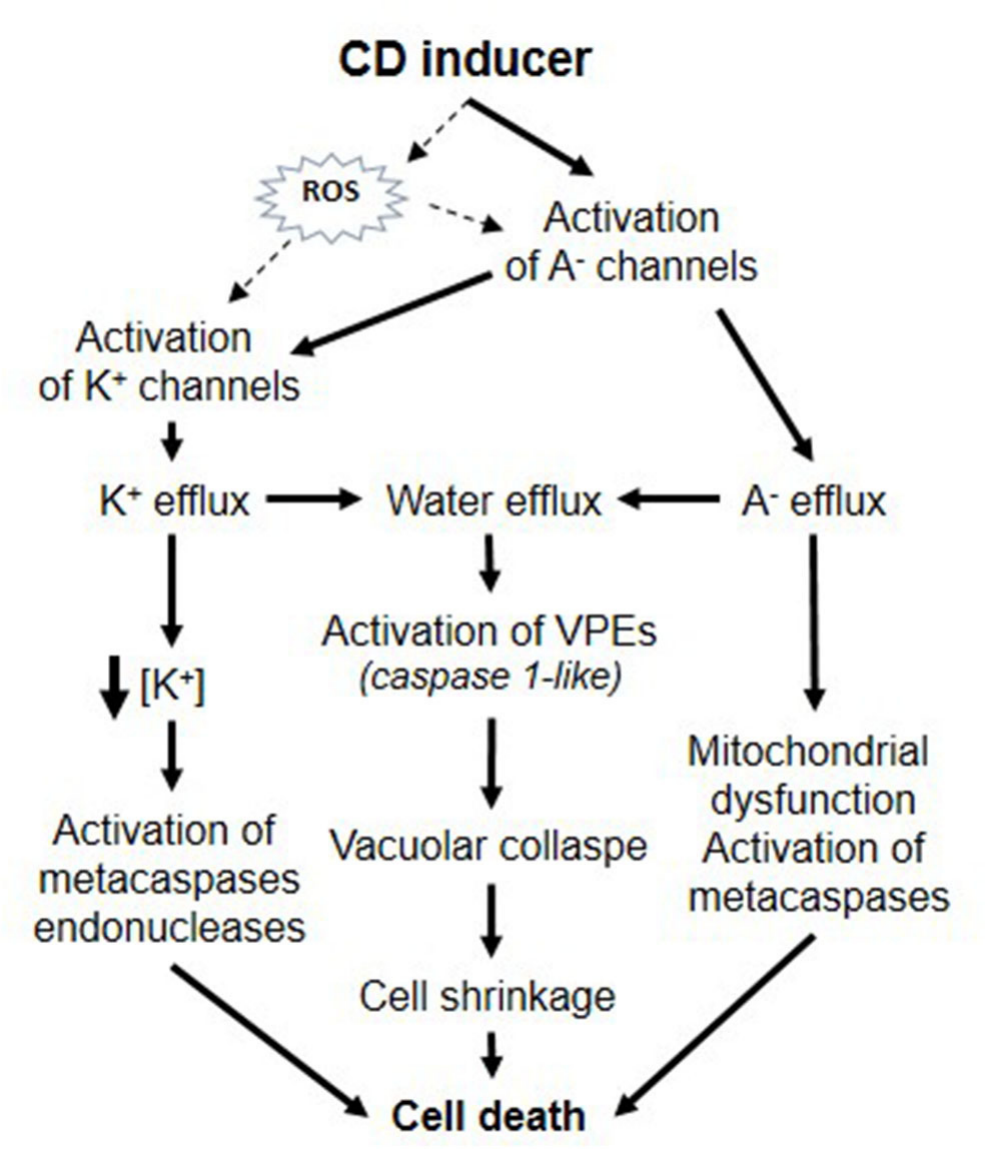
Putative roles of ion channels during plant CD.

As a whole, these data are reminiscent of those described in numerous studies in animal cells and show that plant CD could involve specific modifications of ion transporter activities that could be significant and crucial in the successful propagation of CD (Figure 1). Activation of plasma membrane anion channels and KORC lead to solute loss leading to water release and thus, cell shrinkage could be a major hallmark in plant CD similarly to animal apoptosis. In animal cells, the induction of apoptosis volume decrease is attained by a tightly coupled operation between anion and K^+^ channels and is prevented by application of blockers of Cl^−^ or K^+^ channels (Okada and Maeno, 2001). Furthermore, as in animal models, activation of ion channels was found to precede various CD-inducing process like metacaspase activation. Some CD mechanisms in plant and animals appear mechanistically very similar. According to the sin hypothesis (Ameisen, 2002), the origin of the capacity for self-destruction may be very ancient and due to an intrinsic capacity of the cell in inducing self-destruction. The regulation of ionic channels are involved in numerous vital processes, the control of cell metabolism, volume, and permeability in all organisms and could be a conserved target due to their intrinsic potential to lead cell death. Ion channel mediated CD could thus have a deeply rooted origin and have independently evolved in eukaryotic lineages and multicellular plants and animals.

## Author Contributions

FB, DR, DT, and PL conceived and wrote the manuscript. All authors contributed to the article and approved the submitted version.

## Conflict of Interest

The authors declare that the research was conducted in the absence of any commercial or financial relationships that could be construed as a potential conflict of interest.
